# Impact of Dairy Ingredients on Wheat Flour Dough Rheology and Bread Properties

**DOI:** 10.3390/foods9060828

**Published:** 2020-06-24

**Authors:** Mădălina Iuga, Olga Boestean, Aliona Ghendov-Mosanu, Silvia Mironeasa

**Affiliations:** 1 Faculty of Food Engineering, Ştefan cel Mare University, 13, Universităţii Street, 720229 Suceava, Romania; iugamada@yahoo.com; 2 Faculty of Food Technology, Technical University of Moldova, 168, Ștefan cel Mare Blvd., MD-2004 Chișinău, Moldova; olga.boestean@tpa.utm.md (O.B.); aliona.mosanu@tpa.utm.md (A.G.-M.)

**Keywords:** milk, acid whey, dough rheology, bread characteristics

## Abstract

The incorporation of dairy ingredients, such as milk or acid whey in bread, is advantageous considering their functional properties and the positive effects on consumers’ health. The introduction of an ingredient in bread making process requires the evaluation of dough behavior and final product quality. Thus, the influence of water replacement by milk or acid whey on the characteristics of wheat flour dough and bread was studied. Dynamic rheological measurements were performed in order to evaluate the viscoelastic properties of dough. Compared to the control, an increase of the elastic character of dough for samples with milk and a decrease for those with acid whey was observed. The resistance to deformation decreased when water was substituted with more than 25% milk and increased for samples with up to 25% acid whey. Higher maximum gelatinization temperatures were obtained when water was substituted by milk or acid whey. Bread crumb presented higher firmness, lower volume and porosity for samples with dairy ingredients compared to the control, therefore, replacement levels lower than 25% were recommended in order to minimize this negative effect. Bread elasticity, chewiness, resilience, pores density and size were improved at replacement levels lower than 25%, while for the sensory characteristics of the specialty bread, high scores were obtained. These results can be helpful for processors, in order to develop and optimize bread with dairy ingredients.

## 1. Introduction

In recent years, along with identifying the quality of the product which will mostly satisfy consumer’s needs, the challenge for bakers was to use their knowledge about the local raw materials and processing methods to achieve the required quality. Many different types of breads have been made, and new varieties continue to be developed in order to satisfy consumer demands. The addition of dairy ingredients in bread-making results in bread specialties’ (e.g., buns) development, with an enhanced quality due to their chemical composition [1,2]. Furthermore, milk and acid whey are considered accessible from an economical point of view, especially in some countries where these dairy ingredients are available at low costs [3,4], which means that they are used extensively in bakery products. At the same time, the use of liquid whey or milk in bread manufacture will be beneficial for the bakery industry considering their functional properties [5] and will have a positive impact on consumers’ health. Milk proteins have a high biological value and an increased metabolization rate (96%), being composed of casein, albumins and globulins [6]. In addition, during digestion, milk protein, especially casein, presents specific particularities, as it breaks down trough hydrolysis up to aminoacids and/or soluble and assimilable oligopeptides [6,7,8]. These peptides have an important role as vectors in the diet assimilation of various nutrients, such as vitamin A, calcium and iron [6,9]. Milk protein may exert antimicrobial and immunomodulatory functions and can also induce and maintain satiety [10]. Whey proteins such as β-lactoglobulin, α-lactalbumin, lactoperoxidase and lactoferrin are the compounds responsible for whey health-promoting properties [11]. Acid whey is rich in bio-available amino-acids, such as lysine, which are less present in wheat flour, minerals and vitamins, about 40–50% of milk solids and 20% of proteins being retained in whey [12,13,14]. Whey proteins can develop complexes at macro-, micro-, and nano levels with other components, with promising food applications, for example accomplishing the role of vehicle carrying for some bio-compounds, flavors, or nutrients [15]. Grace to these properties, whey protein represents a valuable ingredient in bakery products [16,17].

Dough rheology plays an essential role in baking products’ quality prediction and may give information about mechanical behavior [18]. Dynamic oscillatory measurement is a fundamental approach widely used for structural and fundamental characteristics of wheat flour dough evaluation [19]. In order to better understand the system behavior, rheological tests like creep-recovery are also required [20], to highlight the importance of mixing energy in dough structure development [21]. These tests can also give information about bread volume potential [22] and dough behavior during processing [23]. Dough and bread characteristics suffer modifications when dairy ingredients are added in the technological process. Therefore, it is necessary to consider certain aspects, such as the percentage added, the type of dairy ingredient and the changes that occur during the production process. The influence of dairy ingredients on dough rheology depends on the composition, and particularly on the dairy protein–gluten interaction [24]. The impact of dairy ingredients, such as liquid milk or acid whey addition, on dough rheological properties is poorly studied. Kenny et al. [25] revealed that the addition of dairy proteins (4%) increased dough farinographic stability. Dough development time decreased when whey was incorporated in bread, a negative effect that is due to the weakening of the gluten matrix [26]. With respect to dough behavior during heating, no studies have been made for wheat dough, in which liquid milk or acid whey was incorporated.

Regarding bread quality, Mannie and Asp [27] reported that milk or its derivates addition to bakery products changes their internal structure, enhances certain flavors and modifies crust color. Lactose from dairy ingredients is a sugar that cannot be fermented by yeasts, remaining in dough after fermentation and taking part in the Maillard reactions during baking process which favors crust browning [28,29]. As whey has a higher lactose content than milk, it has a greater effect on crust color than milk [24]. Native whey proteins have been reported to have a depressant effect on bakery products’ specific volume [30].

Previous studies, which revealed the influence of whey or milk powder, concentrates or proteins on dough and bread characteristics, have been conducted [25,31,32], but, to our knowledge, there is a scarcity of reports about the influence of the liquid acid whey and milk on dough viscoelastic behavior using fundamental rheological tests, textural parameters, crumb microstructure, sensory profile, or the physical, textural and sensory parameters of bread samples. Understanding the interactions of whey or milk with other biopolymers is very important in the sense of novel specialty bread properties improvement. Thus, the aim of this study was to investigate the effects of water replacements at different levels by liquid acid whey and milk, on the rheological characteristics of wheat flour dough and bread quality.

## 2. Materials and Methods

### 2.1. Materials

White wheat flour (2019 harvest) was provided from Franzelcom S.R.L., Chișinău, Republic of Moldova. The acid whey and whole milk (3.5% fat) were provided from a local market of Suceava, Romania and kept at 4 °C until bread making.

Flour quality was tested according to the International and Romanian standards: moisture (ICC 110/1), ash (ICC 104/1), wet gluten (ICC106/1), gluten deformation index (SR 90:2007) and falling number (ICC 107/1) were evaluated. The water absorption capacity (at 15% moisture basis) of wheat flour was determined by using a Consistograph (Chopin Technologies, France), according to the method described by Secchi et al. [33], following the AACC approved method 54-50. The evaluation of wheat dough empirical rheological characteristics in terms of dough tenacity—maximum pressure (*P*) representing the peak height (mm), dough extensibility (*L*) indicated by the length (mm) of the alveogram curve, baking strength (*W*), swelling index (*G*) and configuration ratio of the curve (*P/L*) were achieved using the Alveograph instrument (Chopin Technologies, France), according to the SR EN ISO 27971:2015 standard. All measurements were made in triplicate.

Wheat flour proximate composition analysis was as follows on % dry basis: 10.35 ± 0.15 proteins, 0.90 ± 0.05 lipids, 0.48 ± 0.01 ash, 12.95 ± 0.11 moisture, 29.60 ± 0.10 wet gluten and a deformation index (mm) of 4.00 ± 0.00. The alveogram parameters of the flour used showed a *P* value of 85.00 ± 1.41 mm, an *L* value of 62.00 ± 8.49 mm, *W* of 189.5 ± 23.30 10^−4^ J and *P/L* ratio of 1.39 ± 0.21. According to the results obtained, the white wheat flour used in the experiment is of a medium to strong one for bread making, with a low alpha-amylase activity indicated by the high *FN* value (>320 s) [34,35]. Similar results were reported by Mironeasa and Codină [36] for a 650 wheat flour type, while for a 480 flour type, lower values were reported [37]. 

### 2.2. Bread Making

Bread samples were prepared by using the direct method from wheat flour, 2% salt, 4% sunflower oil, 1% sugar, 2% commercial compressed yeast *Saccharomyces cerevisiae* and water (for the control sample C), according to the water absorption capacity of the flour. The sample formulations were obtained by replacing an amount of water with 12.5% (M1), 25% (M2) and 50% (M3) of milk, or 12.5% (W1), 25% (W2) and 50% (W3) of acid whey. The ingredients were mixed at 200 rpm speed for 15 min in a heavy duty mixer (Kitchen Aid, Whirlpool Corporation, USA). Five dough pieces for each sample formulation of 110 g (53.45 ± 0.05% water absorption) were manually modeled in a spherical form and placed in a fermentation chamber (PL2008, Piron, Italy) for 180 min at 30 °C and 85% relative humidity. The bread was baked in an oven (PF8004D, Piron, Italy) for 15–20 min at 220 °C. After cooling for 3 h, the samples were subjected to analysis.

### 2.3. Dough Rheological Properties

The fundamental rheological characteristics of dough were assessed by the dynamic oscillatory method in the linear viscoelastic region. For this purpose, a HAAKE MARS 40 stress controlled rheometer (Thermo-HAAKE, Karlsruhe, Germany) with smooth parallel plates measuring system and a gap width of 2 mm were used. The dough samples were formulated without yeast, at the optimum water absorption capacity previously determined. Dough formulations rested for 5 min to allow relaxation and temperature stabilization, then were placed between the plates and kept for 120 s prior testing. The measuring temperatures were controlled by using an external thermostatic bath during the tests. The excess of dough was removed, and a Vaseline layer was applied to the exposed edge of dough, to prevent the loss of moisture during testing. Before analysis, dough samples were tested for the limits of the linear viscoelastic region (LVR) based on the strain sweep determination, in which the strain was increased from 0.01 to 1%, at a constant oscillation frequency of 1 Hz [38]. The oscillatory measurements were performed at a maximum strain of 0.15%, which was found to be in the LVR where dough samples have a linear relationship between stress and strain.

#### 2.3.1. Frequency Sweep Test

The frequency sweep test was performed at a frequency variation from 1 to 20 Hz, with a constant stress previously established, in the LVR, at a temperature of 20 °C. The changes of the storage modulus (*G′*), loss modulus (*G″*) and complex modulus (G*) were registered. The experimental data were fitted to the Power law model using the Equations (1)–(3) [38,39]. The loss tangent (*tan δ*) was calculated as the ratio between *G″* and *G*’.

(1) $$G^{\prime }\left(\omega \right)=K^{\prime }\cdot \omega^{n^{\prime }}$$

(2) $$G^{\prime \prime }\left(\omega \right)=K^{\prime \prime }\cdot \omega^{n^{\prime \prime }}$$

(3) $$G^{*}\left(\omega \right)=K^{*}\cdot \omega^{n^{*}}$$

where *G*′ is the storage modulus (Pa), *G*″ loss modulus (Pa), G* complex modulus (Pa), *ω* angular frequency (rad/s), *K′*, *K″*, K* (Pa·s^n′^) are consistency indices, *n′, n″* and n* are flow behavior indices.

#### 2.3.2. Creep and Recovery Test

Creep-recovery tests were performed by applying a constant shear stress of 50 Pa over a creep time of 60 s and allowing strain recovery during 180 s after stress removal, at 20 °C temperature. The compliance rheological parameter [38,40] was recorded (Equation (4)):

(4) $$J\left(t\right)=\frac{\gamma \left(t\right)}{\sigma }$$

where *J* (Pa^−1^) is the compliance, *γ* the strain and *σ* the constant stress applied (Pa^−1^). 

The experimental data of creep-recovery test were fitted to the Burgers model, made up of four components, which comprises the association in series of the Maxwell model and the Kelvin–Voigt model [40,41]. Equation (5) shows the Burger model during creep phase and Equation (6) during the recovery phase:

(5) $$J\left(t\right)=J_{Co}+J_{Cm}\left(1-\exp\frac{-t}{\lambda_{C}}\right)+\frac{t}{\mu_{Co}}$$

(6) $$J\left(t\right)=J_{max}-J_{Ro}-J_{Rm}\left(1-\exp\frac{-t}{\lambda_{R}}\right)$$

where *J_Co_*, *J_Ro_* (Pa^−1^) are the instantaneous compliances for creep and recovery phase, respectively, *J_Cm_* and *J_Rm_* (Pa^−1^) are the retarded elastic compliances or viscoelastic compliances for creep an recovery phase respectively, *t* (s) is the phase time, *λ_c_*, *λ_R_* (s) are the retardation times, *μ_Co_* (Pa·s) is the zero shear viscosity and *J_max_* (Pa^−1^) is the maximum creep compliance obtained at the end of the creep test. The recovery compliance, *J_r_* (Pa^−1^), evaluated when dough recovery reached equilibrium, is calculated by the sum of *J_Ro_* and *J_Rm_*. 

The percentage recovery, which highlighted the relative elastic part of the maximum creep compliance, was also determined as the ratio between *J_max_* and *J_r_* [38].

#### 2.3.3. Temperature Sweep Test

The temperature sweep test was performed at a constant strain of 0.15% and a frequency of 1 Hz, the dough samples being heated from 20 to 100 °C at a rate of 4.0 ± 0.1 °C per min. The storage (*G′*) and loss modulus (*G″*) were recorded as a function of temperature, allowing the determination of maximum gelatinization temperatures (*T_max_*).

### 2.4. Bread Physical Properties

The physical properties of bread in terms of specific volume (determined by seed displacement method), porosity and elasticity were evaluated in triplicate, according to the Romanian standard SR 91:2007. Crumb cylinders of 45.50 diameter and 60 mm height were weighted, and their volume was measured in order to determine bread samples porosity (Equation (7)). For crumb elasticity achievement, the cylinders height was measured, then they were pressed until half of their height for 1 min, released and relaxed for 1 min and the final height was recorded. The difference between the initial measurement and the height after pressing and relaxing represents elasticity [42].

(7) $$Porosity\;\left(\%\right)=\frac{V-\frac{m}{\rho }}{V}\cdot 100$$

where, *V* represents crumb cylinder volume in cm^3^, m is the crumb cylinder weight in g, and *ρ* is the crumb density in g/cm^3^.

### 2.5. Texture Profile Analysis

The texture profile analysis (TPA) was performed using a TVT-6700 texture analyzer (Perten Instruments, Hägersten, Sweden). Bread sample slices of 50 mm in height were submitted to two cycles of compression up to 20% of the original height using a 45-mm cylindrical probe, at a speed of 1.0 mm/s, a trigger force of 5 g and a recovery period between compressions of 15 s. The parameters determined were firmness, cohesiveness, gumminess, chewiness and resilience. Four measurements were done for each sample.

### 2.6. Crumb Microstructure 

The crumb microstructures were achieved by using a MoticSMZ–140 (Motic, Xiamen, China) stereo microscope with a 20× objective. For this purpose, the bread samples were cut into slices of 10 mm. Three images per sample were recorded at a resolution of 1024 × 768 pixels and processed using ImageJ software (ImageJ 1.52a version, National Institutes of Health, USA), for 1 pixel; the corresponding distance being 12.6 μm. Each image was converted into an 8-bit grayscale and the Otsu algorithm, as a threshold method for gas cells differentiation, was used [43]. The shapes larger than 0.01 mm^2^ were considered gas cells, as the human eye can perceive particles with approximately 0.1 mm [44]. 

### 2.7. Sensory Profile 

The sensory profile of the bread samples was evaluated in duplicate, in two sessions, by a panel of 13 semi-trained judges. The evaluated characteristics were: appearance, color, flavor, texture, taste, smell, texture and overall acceptability using a 9-point scale. A score of 1 represented “extremely dislike”, a score of 5, “neither like nor dislike” and a score of 9 represented “extremely like”.

### 2.8. Statistical Analysis

The statistical software SPSS 26.0 (trial version) for Windows (IBM, New York, NY, USA) was used for data processing. The results were expressed as mean value ± standard deviation. An analysis of variance with one factor (one-way ANOVA) was applied in order to evaluate the differences between means by using Tukey’s test at a 5% significance level. Statistically significant differences were considered at *p* < 0.05. A principal component analysis (PCA) was carried out, in order to evaluate the relationships among the evaluated characteristics and to visualize the similarities between them. 

## 3. Results 

### 3.1. Dough Rheological Properties

#### 3.1.1. Frequency Sweep Test

**Figure 1:**
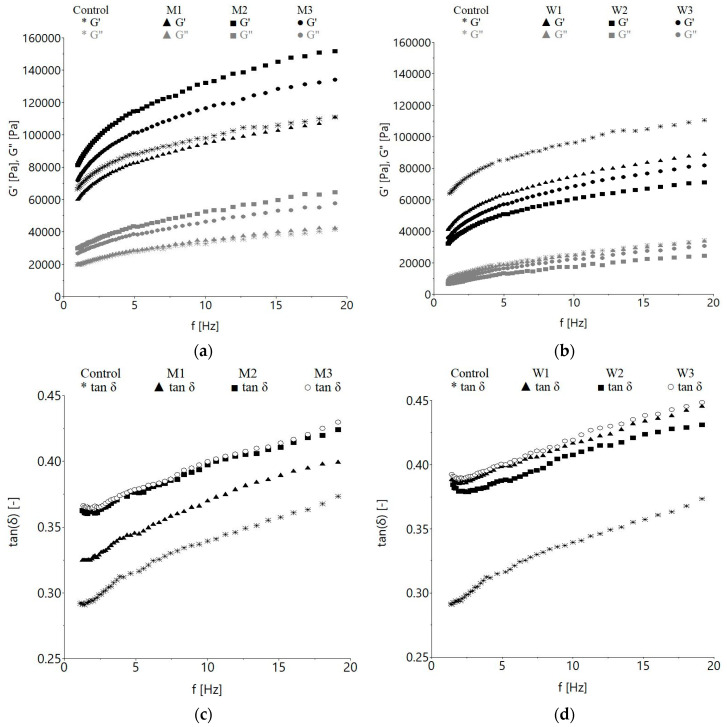
Storage (*G′*) and loss modulus (*G″*) variations with frequency for: (**a**) milk containing samples, (**b**) acid whey containing samples. Loss tangent (*tan δ*) variation with frequency for: (**c**) milk containing samples, (**d**) acid whey containing samples. M1—12.5% milk, M2—25% milk, M3—50% milk, W1—12.5% acid whey, W2—25% acid whey, W3—50% acid whey.

The frequency sweep tests data obtained showed that, in the range of the considered frequencies, the mechanical spectra of the storage modulus (*G′*) was greater than of the loss modulus (*G″*) for the control, the samples with milk and those with acid whey, indicating that the dough have a viscoelastic behavior, as was expected (Figure 1). 

The tested samples exhibited a predominant elastic solid-like behavior (*G′* > *G″*) over the entire experimental frequency range. The *G′* and *G*″ moduli increased with the frequency increase, which means that dough recovery after a stress application was slow, due to the fact that the network is not completely elastic. The replacement of water with milk led to a significant (*p* < 0.001) increase of storage and loss moduli compared to the control, except for M1—the sample with the lowest replacement level (Figure 1a), whereas a significant (*p* < 0.001) decrease of the *G′* and *G″* values at 10 Hz with a percentage increase from 12.5 to 50% was found when acid whey replaced water (Figure 1b). Significant differences (*p* < 0.001) were observed in loss tangent (*tan δ)* at 10 Hz, for the control and dairy ingredient-containing dough (Figure 1c,d). The increase of *tan δ* was proportional with the frequency increase for all the tested samples. Compared to the control, the samples with milk or acid whey presented significantly (*p* < 0.001) higher values of *tan δ.* Milk containing samples presented significantly (*p* < 0.001) lower *tan δ* compared to those with acid whey. Among the dough samples with milk, the highest values were observed for M2 and M3, while among those with acid whey, W3 had greater *tan δ* values compared to W1 and W2, the differences among samples being significant (*p* < 0.001). 

The viscoelastic moduli variation with frequency was fitted by the Power law model (Equations (1)–(3)); the values of *K′*, *n′*, *K″*, *n″*, K* and n* parameters corresponding to *G′, G″* and G* are shown in Table 1. The coefficients of determination (*R^2^*) values were higher than 0.999, 0.985 and 0.999 for *G′, G″* and G* respectively, which indicates a very good model fitting. Related to dough consistency, the coefficients *K′*, *K″* and K* from the Power law model reflect the magnitude of viscosity in terms of consistency [45]. As can be depicted from the results obtained, the values of *K′*, *K″* and K* significantly (*p* < 0.001) increased for milk-containing samples compared to the control, except for M1, while for the dough with acid whey *K′*, *K″* and K* significantly (*p* < 0.001) decreased with the concentration increase (Table 1); W2 and W3 samples being affected to the greatest extent. 

#### 3.1.2. Creep and Recovery

**Figure 2:**
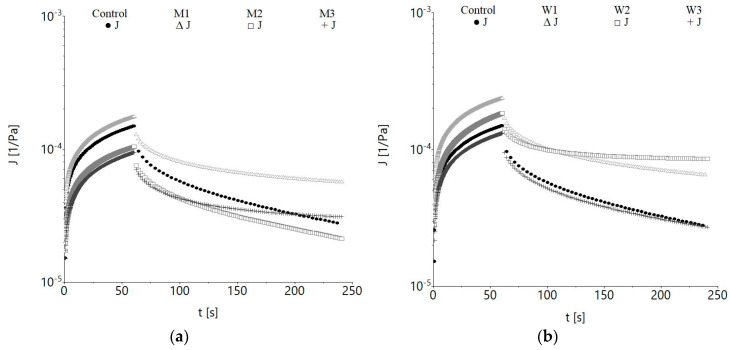
Creep and recovery curves of wheat flour dough at different water replacement levels by: (**a**) milk: M1—12.5%, M2—25%, M3—50%, (**b**) acid whey: W1—12.5% W2—25% W3—50%.

According to the results presented in Figure 2, the replacement of water by milk or whey clearly influenced the viscoelastic properties of doughs. 

Milk doughs with more than 25% (M2 and M3) showed significantly (*p* < 0.001) lower compliance values during the creep test (Figure 2a). Significantly (*p* < 0.001) higher compliance values for M1 (12.5% milk) indicate a higher deformability than M2, M3 and the control. The replacement of water by acid whey determined a significant (*p* < 0.001) increase of the resistance to deformation of W1 and W2, while for W3, the resistance to deformation decreased compared to the control (Figure 2b).

The results of the creep and recovery tests were satisfactorily fitted to the Burgers model, always yielding values of *R*^2^ ≥ 0.913, in order to evaluate the differentiation of the samples by the effect of water replacement with dairy ingredients (Table 2). The control and milk added products doughs exhibited different patterns of behavior. Compared to the control sample, M1, W1 and W2 doughs are characterized by significantly higher (*p* < 0.001) values of the *J_Co_* parameter, which characterize the instantaneous elasticity, indicating a firmness improvement of the bread doughs’ structure. *J_Cm_* values decreased compared to the control for the samples containing milk (*p* < 0.001), except M1, and increased for those with acid whey, except W3. A proportional decrease of *J_Co_* and *J_Cm_* with the replacement level increase was observed for both milk and acid whey containing samples. Acid whey substituting water at 12.5% level (W1) resulted in creep phase instantaneous compliance (*J_Co_*), retarded elastic compliance (*J_Ro_*) and compliance in the recovery phase (*J_r_*) increased significantly (*p* < 0.001) with respect to the control dough values. A significant (*p* < 0.001) *J_Ro_* increase was observed for M1 and M3 compared to the control. The replacement of water with milk or acid whey from 12.5 to 50% led to a significant decrease (*p* < 0.001) of *J_Rm_* with the level increase (Table 2), except W3, which slightly increased. Compared to the control, a significantly (*p* < 0.001) higher retardation time for the creep phase *λ_C_* was obtained for all samples, except W1, which is similar to the control (*p* > 0.05), while for the recovery phase, *λ_R_* value was greater only for M2. Dough flowability at the end of the applied load described by *μ*_0_ was non-proportionally influenced by the replacement level for both milk and acid whey containing samples (Table 2). Lower flowability was found for M2, M3 and W3 dough samples, compared to C, indicating an increase of dough strength. 

A significant (*p* < 0.001) increase of *J_max_* and *J_r_* was obtained for M1, W1 and W2, while in M2, M3 and W3 *J_max_* decreased significantly (*p* < 0.001) compared to the control. The results showed a significant (*p* < 0.001) decreasing trend of *J_max_* and *J_r_* among milk (M1-M3) or acid whey (W1-W3) containing samples with the replacement level increase. Higher *J_max_* values, in increasing order, were obtained for W1, W2 and W3, compared to milk containing samples. The *J_r_/J_max_* ratio values increased compared to the control and ranged between 65.43 and 73.95%. Significant differences (*p* < 0.001) of *J_r_/J_max_* were obtained only between samples with acid whey and control (Table 2), the values close to those of the wheat flour indicating a well-developed structure [46]. Among dairy ingredients, the highest *J_r_/J_max_* ratio was obtained by the sample with 50% milk and with 25% acid whey, the elasticity of the last one being higher.

#### 3.1.3. Temperature Sweep Test

**Figure 3:**
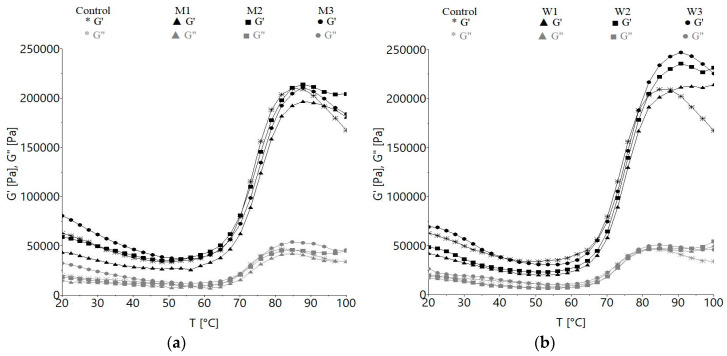
Effect of water replacement by: (**a**) milk: M1– 12.5%, M2—25%, M3—50%, (**b**) acid whey: W1—12.5% W2—25% W3—50%, on the changes of storage modulus (*G′*) and loss modulus (*G″*) vs. temperature.

The changes of the viscoelastic moduli with temperature are showed in Figure 3. At the beginning, the decrease of *G′* to a minimum up to a certain temperature occur, then the *G′* modulus abruptly increase until achieves the maximum gelatinization temperature, then it decreases again. The samples containing acid whey or milk exhibited higher gelatinization temperatures compared to the control (Figure 3). The peak values of *G′* and *G″* were significantly (*p* < 0.001) higher for milk and acid whey containing samples, except for M1. The storage modulus values began to increase rapidly between 48.10 and 56.22 °C, reaching a peak between 84.79 and 93.84 °C (Table 2), depending on the type and amount of ingredient, and then slowly decreased (Figure 3). 

The maximum gelatinization temperatures (*T_max_*) of all assayed samples are shown in Table 2. An increase of the maximum gelatinization temperatures (*T_max_*) compared to the control was obtained, revealing significant influences (*p* < 0.001) of milk or acid whey. 

### 3.2. Bread Physical Properties

Bread physical properties in terms of volume, porosity and elasticity are among the first characteristics subjectively evaluated by consumers when choosing the desired product. The effect of water replacement by milk and acid whey is shown in Table 3. 

The specific volume of all the tested samples were significantly (*p* < 0.001) lower compared to the control. The specific volume of bread decreased with the replacement level increase for milk containing samples, a similar trend being observed for those with acid whey, but the decrease did not follow a specific pattern. The decrease in bread specific volume was more accentuated for the samples with milk compared to those with acid whey. 

The porosity of the studied samples was significantly (*p* < 0.001) lower compared to the control, the decrease being more accentuated for the samples with milk than those with acid whey. 

With respect to bread elasticity, milk containing samples presented significantly (*p* < 0.001) lower values compared to the control, exceptM2 (Table 3), while the inclusion of acid whey led to higher values (*p* < 0.001). 

### 3.3. Texture Profile Analysis

Bread texture characteristics directly influence consumer perception and choice. The results of the texture profile analysis are shown in Table 4. 

A significant (*p* < 0.05) increase of firmness, gumminess, chewiness and resilience for samples with milk or acid whey compared to the control was observed (Table 4). Crumb cohesiveness significantly increased (*p* < 0.05) for the samples with milk compared to the control, while for those with acid whey, it decreased. Gumminess values are significantly (*p* < 0.001) higher compared to those of the control, but an irregular trend with the replacement level was observed for both milk and acid whey containing samples. M2 and W2 samples exhibited the highest firmness, gumminess and chewiness, while M1 and W1 presented the highest cohesiveness.

### 3.4. Crumb Microstructure

**Figure 4:**
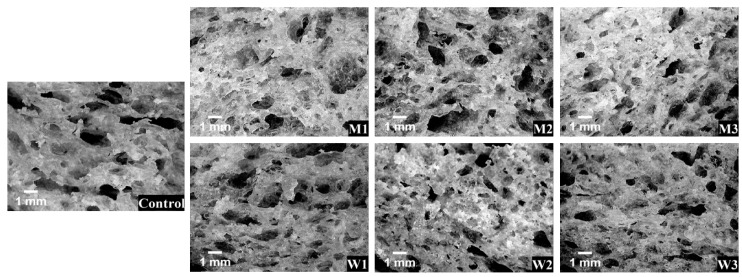
Crumb microstructure of bread samples with different water replacement levels: M1—12.5% milk, M2—25% milk, M3—50% milk, W1—12.5% acid whey, W2—25% acid whey, W3—50% acid whey.

The replacement of water by milk or acid whey led to changes of crumb microstructure, in terms of pores density, size, form and area (Figure 4). The values of pores density significantly (*p* < 0.001) increased for milk containing samples, except M1, for which it decreased, compared to the control (Table 5). On the other hand, whey containing samples exhibited a decrease of pores density compared to the control, except W1, for which no significant difference (*p* > 0.05) was observed. The pores density significantly (*p* < 0.001) increased with the replacement level increase for the milk containing samples, while for those with acid whey, it decreased (*p* < 0.001). 

Compared to the control sample, M2 and M2 presented higher pores sizes, while lower values were obtained for W2 and W3. Pores circularity and cell area fraction did not differ significantly (*p* > 0.05) among samples for both replacements. 

### 3.5. Sensory Profile

**Figure 5:**
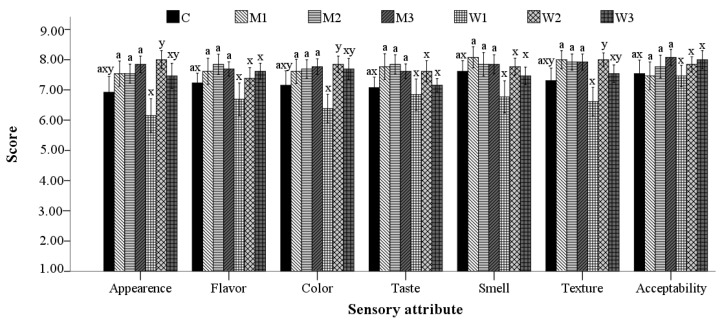
Sensory characteristics of bread samples with different water replacement levels by: milk: M1–12.5%, M2—25%, M3—50% (a, mean values followed by the same letter are not significantly different (*p* > 0.05)); acid whey: W1—12.5% W2—25% W3—50% (x and y, mean values followed by different letters are significantly different (*p* < 0.05)).

The sensory characteristics evaluated for specialty bread with milk and acid whey, respectively, are shown in Figure 5. Compared to the control, no significant differences (*p* > 0.05) in terms of appearance, flavor, color, taste, smell, texture or acceptability were obtained. Bread appearance, color and texture scores were significantly different (*p* < 0.05) between W1 and W2 samples, while no significant differences (*p* > 0.05) among the samples with milk were observed. The replacement of water with 12.5% acid whey (W1) led to the lowest scores for all the attributes evaluated, while W2 obtained the best scores, except for overall acceptability.

### 3.6. Correlations Analysis between Dough Rheology and Bread Characteristics

Using Pearson correlation analysis, a range of correlation coefficients (*r*), which varied between 0.46 and 0.99, was found between the rheological dough parameters and bread characteristics, significance at *p* < 0.05 level (Table 6). The specific volume parameter is positively correlated (*r* = 0.50; *p* < 0.05) with the recovery compliance (*J_max_*) and *J_Co_* (*r* = 0.50; *p* < 0.05), negative interrelations being observed with the consistency indices *K″* (*r* = −0.63; *p* < 0.01) and K* (*r* = −0.45; *p* < 0.05) and creep parameters *λ_C_* (*r* = −0.46; *p* < 0.05)*, μ_Co_* (*r* = −0.54; *p* < 0.05). Significant positive correlation at *p* < 0.01 was obtained for crumb firmness, with flow behavior indices *n′* (*r* = 0.75) and n* (*r* = 0.61). A significant correlation at *p* < 0.01 was found between the maximum gelatinization temperature, *T_max_*, and bread firmness (*r* = 0.64). 

Significant correlations were also obtained between the rheological parameters tested at small and large deformations. The shear viscosity, *μ_Co_*, presented significant high correlation with consistency indices *K′* (*r* = 0.74; *p* < 0.01) and *K″* (*r* = 0.83; *p* < 0.01). Consistency index *K′* showed a significant negative interrelation with instantaneous compliances *J_Co_* (*r* = −0.58; *p* < 0.01) and *J_Ro_* (*r* = −0.68; *p* < 0.01), with the retarded elastic compliance *J_Cm_* (*r* = −0.71; *p* < 0.01) and with the recovery compliance *J_r_* (*r* = −0.70; *p* < 0.01). Consistency index *K″* was negatively correlated with instantaneous compliances *J_Co_* (*r* = −0.60; *p* < 0.01) and *J_Ro_* (*r* = −0.62; *p* < 0.01), with the retarded elastic compliance *J_Cm_* (*r* = −0.61; *p* < 0.01), and with the recovery compliance *J_r_* (*r* = −0.70; *p* < 0.01). As expected, high positive correlations at *p* < 0.01 were found between creep compliance *J_Co_* and the recovery parameters *J_Ro_* (*r* = 0.70)*, J_Rm_* (*r* = 0.88), *J_r_* (*r* = 0.95), *J_Cm_* also being correlated with *J_Ro_* (*r* = 0.87; *p* < 0.01), *J_Rm_* (*r* = 0.52; *p* < 0.05), *J_r_* (*r* = 0.89; *p* < 0.01), since the creep-recovery tests were carried out in the LVR (data not shown). Furthermore, viscosity at the steady state (*μ_Co_*) increase led to a decrease of *J_max_, J_Ro_, J_Rm_* and *J_r_* compliances values (*p* < 0.01). Pores density was found to be associated with the creep compliances *J_Ro_* (*r* = −0.57; *p* < 0.01) and *J_r_* (*r* = −0.46; *p* < 0.05). Significant correlations at *p* < 0.01 were found between the maximum gelatinization temperature, *T_max_*, and K* (*r* = −0.69), n* (*r* = 0.72), *J_Cm_* (*r* = 0.71), *J_max_* (*r* = 0.57), *J_Ro_* (*r* = 0.56) and *J_r_* (*r* = 0.60).

### 3.7. Multivariate Analysis of Bread Characteristics

**Figure 6:**
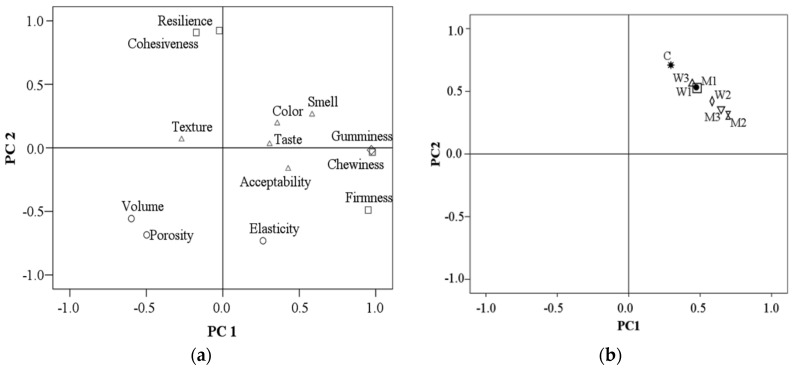
Principal component analysis: (**a**) distribution of the physical, textural and sensory parameters analyzed; (**b**) distribution of bread samples in function of the analyzed parameters. C—control sample, M1—12.5% milk, M2—25% milk, M3—50% milk, W1—12.5% acid whey, W2—25% acid whey, W3—50% acid whey.

The principal component analysis (PCA) was applied in order to reveal the relationships between bread samples analyzed variables. The relationships between physical, mechanical textural and sensory characteristics of bread samples analyzed are shown in Figure 6a. The first two principal components explain 58.99% of the total variance (*PC1* = 35.92% and *PC2* = 23.07%). The *PC1* was associated with chewiness, gumminess, smell, porosity, volume and firmness, while *PC2* was associated with elasticity, resilience and cohesiveness. Significant correlations at 0.01 level were obtained for porosity with volume (*r* = 0.88), gumminess with volume (*r* = 0.64) and porosity (*r* = 0.61), chewiness with volume (*r* = 0.63) and porosity (*r* = 0.60), resilience and porosity (*r* = 0.67) and acceptability with color (*r* = 0.71). The *PC1* highlights an opposition between cohesiveness and porosity, color and acceptability, while *PC2* underlines oppositions between resilience and elasticity, cohesiveness and elasticity, volume and firmness.

The distribution of all bread samples in the function of the physical, textural, and sensory characteristics is presented in Figure 6b. The first two components explain 99.96% of the total variance (*PC1* = 99.90% and *PC2* = 0.06%). Significant correlations at 0.01 levels among all the bread samples were obtained. Clustering of the samples can indicate the similarity between the interrelated physical, textural and sensory parameters. Based on the bi-plot of the principal component scores, the control sample was quite distinct from all the other samples (Figure 6b), suggesting significant differences among the considered parameters. 

## 4. Discussion

### 4.1. Dough Rheological Properties

The rheological tests may give information about dough behavior during processing stages. Furthermore, information about the functional roles of some chemical constituents of dairy ingredients which can inhibit or promote some molecular interactions in the dough system can be achieved [47]. All the tested samples exhibited a solid-like behavior, the *G′* and *G*″ moduli increasing with the frequency. Similar behaviors were reported for wheat dough with different water levels, dough enriched with whey concentrate, lactose dairy powder, yoghurt or curd cheese [48,49,50]. The decrease of the viscoelastic moduli as the amount of acid whey was increasing was probably due to the cutting effect of the large amount of whey components, which can disturb the structure and intensity of the continuous gluten network during dough formation. A synergistic effect was found for the rheological behavior of starch–caseinate mixtures, which showed higher apparent viscosities than did the single component, as reported by Lelievre and Husbands [51]. The *tan δ* provides information about the structural order (molecular interactions) of a material, highly structured materials having low *tan δ* values [52]. An increasing tendency proportional with the frequency increase for all the tested samples was obtained, indicating the predominance of viscous characteristics in dough and decrease of the elastic characteristics at higher frequencies. These results denote that the capacity of the tested dough sample for dissipation, as measured by the *G″* value and storage, as measured by the *G′* of the energy used for its deformation increase with the oscillation frequency increase [53]. The replacement of water with acid whey or milk led to higher *tan δ* compared to the control. This may be due to the increase of the polymers amount in dough system which led to higher elastic properties, similar with the data reported by Nammakuna et al. [54] for wheat-rice dough with whey protein isolates. It was demonstrated that low *tan δ* values indicate rigid and stiff dough, while higher *tan δ* values are related to a moist and slack structure [55]. Higher *tan δ* for the samples with 50% replacement level by dairy ingredients can probably be caused by the water-soluble solids which determine greater *tan δ* values [56]. Therefore, substituting water in the control dough with 12.5% (M1) milk led to the lowest *tan δ* compared to M2 and M3, while the replacement with 25% (W2) whey determined the lowest values among whey containing samples. It could be said that, at these levels, the substitutions induce a change of dough network structure, conferring greater structural stabilization compared to the other samples. The high elasticity in milk containing doughs, except M1, across the test range of frequencies could be associated with the presence of milk components in high amounts in the formulation. Milk components may increase the number of hydrogen bonds with water, increasing the water retention capacity and providing structural stabilization. Water substitution with 25% acid whey (W2) led to the greatest decrease of consistency coefficient compared to the control sample, which is a measure of the shear viscosity. However, acid whey doughs had similar behavior, the results confirming the slight weakening of dough. It seems that acid whey components worked as inert ingredients and did not interact with other dough components, since lower responses of *G′* and *G″* were obtained for the samples containing acid whey compared to the control dough. The consistency indices (*K′*, *K″* and K*) were strongly negatively correlated (*p* < 0.01) with the maximum gelatinization temperature *T_max_* (Table 6). The flow behavior index *n′* was significantly influenced (*p* < 0.001) by the addition of milk or acid whey compared to the control. Acid whey-containing doughs had shown a more prominent effect on the consistency coefficients compared to those with milk at 25% and 50% substitution levels. Overall, water replacement by acid whey significantly reduced storage modulus and showed less shear thinning behavior compared to milk containing samples. This fact could be due to less starch granules swelling in the presence of acid whey and reduced amylose leaching, resulting in lower viscosity. Similar findings were reported by Kumar et al. [57] when oat starch was substituted with skim milk powder, whey protein concentrate and whey lactalbumin. The *n′, n″* and n* parameters can be related to the relaxation exponent (*n_r_*) and true gels should have *n′* = *n″* = *n_r_*. Gels with *n_r_* values close to 1 are purely viscous, whereas *n_r_* close to 0 indicates a purely elastic gel [52]. Table 1 shows that *n′* and *n″* were higher than 0.176 (n* value of control sample) in the case of either milk or whey containing samples, suggesting that the doughs had a strong viscous character. The relative magnitudes obtained for *n′* and *n″* are typical of weak physical gels, being comparable to those determined for other dairy ingredients incorporations, such as skim milk powder, whey protein concentrate and whey lactalbumin on oat starch properties [57]. Higher storage modulus of dough with milk compared to those of the samples with acid whey can be attributed to the starch grain rigidity increase induced by lactose or to the preferential absorption of it by the swollen starch grains [58].

Doughs with more than 25% milk (M2 and M3) had lower compliances during the creep stage (Figure 2a), indicating lower deformability than the control sample, and thus a stronger matrix structure, according to Tarancón et al. [59]. Laguna et al. [60] state that smaller compliances are indicative of a stronger dough matrix which exhibits increased resistance to deformation. The obtained results suggest that the addition of milk reinforced the dough structure (higher values for the Burger model parameters and *G′* and *G″* were obtained) and presented greater resistance to deformation compared to the control. Conversely, significantly (*p* < 0.001) higher compliance values for M1 (12.5% milk) indicate a higher deformability than M2, M3 and control. Higher milk dough deformability can be explained by the hydrogen bonds, which are physical linkages and can break and recombine [59]. An increase of dough with more than 25% milk rigidity suggested the presence of an end-linked network of glutenin infiltrated by gliadin polymers. An increased viscosity of the system will contribute to the aggregation of polymers increase, concomitant to the repulsion forces between polymers decrease caused by milk components. A similar finding was reported by Chompoorat et al. [61] when investigating the effect of diacetyl tartaric acid ester of monoglycerides (DATEM) on the viscoelasticity of wheat gluten, which caused a gluten rigidity increase by promoting the protein-starch interactions. Higher compliance values for W1 and W2 compared to the control can be due to the casein micelles stabilization by means of electrostatic repulsions; greater acidities leading to a smaller stabilization effect [62]. In the case of W3, the rougher surface of casein micelles probably do not allow neutral polymers from binding on them and calcium ionic interactions, which can partially substitute the disulphide bonds’ behavior, leading to rheological parameters’ values close to that of the control (Figure 2b) [62,63]. Creep phase instantaneous (*J_Co_*), retarded elastic compliance (*J_Ro_*) and compliance in the recovery phase (*J_r_*) values were higher for W1 compared to the control, indicating that whey dough with a 12.5% level had higher instant and retarded deformation when it was subjected to a constant stress, and higher recoveries when the stress was removed. A greater instantaneous and retarded elastic compliance can be related to the dough water content rising when the hydrocolloid is absent [64]. A more compliant elasticity during recovery phase (*J_Ro_*) was obtained for W1 and W2 samples compared to C, suggesting greater recoverable energy stored by a more cross linked gluten, due to the higher number of disulfide bonds compared to the control, according to Chompoorat et al. [61] Significant negative strong correlations (*p* < 0.01) were found between *J_max_, J_Co_, J_r_* and *J_Ro_* respectively (Table 6) and consistency coefficients (*K′, K″* and K*) of the Power law model. This means that lower compliances that suggest smaller deformability led to higher consistency indices, which indicate system rigidity increase, similar to the results reported by Laguna et al. [60] The significant (*p* < 0.01) negative correlation obtained between the consistency indices and the recovery compliances may suggest that dough with high recovery capacity presents a lower consistency. Positive correlations (*p* < 0.01) were obtained for the maximum gelatinization temperature (*T_max_*) with Burgers parameters for the creep stage *J_Co_*, *J_Cm_, μ_Co_* and for the recovery stage respectively *J_Ro_* and *J_r_* (Table 6). The increase of *J_Ro_* in M1 and M3 compared to the control can be explained by the ability of acid whey compounds to change protein conformations. The increase of *J_Rm_* when water was replaced by milk or acid whey means lower instant and retarded deformations when a constant stress was applied and smaller recoveries when the stress was removed [38]. In respect to the retardation time, similar values with those reported by Mironeasa et al. [38] were found. Higher *J_max_* for the samples with acid whey compared to those with milk can probably be due to the amount of available water in the dough samples. According to Mastromatteo et al. [65] the creep compliance increases proportionally with water contents at constant stress increase, whereas the elastic contribution decreases. The greater recovery of milk or acid whey containing samples given by the increased *J_r_/J_max_* ratio means enhanced recuperation capacity, which may be useful in dough handling. The recovery ratio may give some information about dough macrostructure. For instance, higher recovery values may be given by the presence of small molecules, while lower values may be related to the large molecules [66]. Furthermore, a higher *J_r_/J_max_* ratio led to better dough film resistance to rupture between pores [21], the elasticity being essential in order to prevent gas cells destruction under gravity [67]. Creep and recovery parameters correlations obtained are in good accordance with previous observations [68,69]. 

During heating, storage modulus (*G′*) and viscous modulus (*G″*) showed different behavior with the increase of temperature. In the first stage, the gradual decrease of *G′* to a minimum up to a certain temperature is the result of protein denaturation which affects their water absorption capacity, and indicates dough softening [70]. Then, due to the gelatinization process that occurs around 50 °C, the *G′* modulus abruptly increased until it achieved the maximum gelatinization temperature, followed by a decrease. The abrupt increase of *G′* is related to the starch grains swelling and distortion, as they act as fillers in the gluten network and promote effective cross-linkages [71]. Dough viscosity and elasticity increased due to the starch gelatinization process and proteins’ interactions, and was finished in the ultimate stage, when the *G′* and *G″* began to decrease. A similar trend was reported by Zhou et al. [72] for whey protein enriched bread dough. Higher *G′* and lower *G″* values can be due to the protein-protein interactions, which tend to determine an increasingly cross-linked structure [73]. *G′* increase during heating has been reported to be proportional with dough starch content, and thus, the modifications during heating are related to the changes of starch structure [74]. Gelatinization temperature changes have also been obtained for doughs with hydrocolloids added, probably as a result of their interaction with starch, which determines variations of the gelatinization temperatures, depending on the hydrocolloids type and concentration [75]. The replacement of water with milk or acid whey led to higher maximum gelatinization temperature compared to the control, similar results being reported by Tang and Liu [76] for whey enriched protein dough, probably due to the dairy proteins’ interactions with wheat components. It is possible that a matrix is formed through hydrogen bonds behind the interactions of dairy proteins with swollen starch grains, soluble starch components leached from the grain during heating and damaged starch granules [77]. The significant effect of milk in delaying the starch gelatinization can be related to the water substitution with milk in dough, which can increase the energy necessary for chemical and physical reactions involving water [78]. Regarding this, the acid whey and the proteins content can contribute to the increase of the sites for cross-linking among proteins and starch grains, while synergistic interactions between whey proteins and starch may occur [58,76]. The differences between the samples with milk and acid whey can be explained by the ability of whey proteins to form gels [72], the influence of ions and lactose within milk protein also being important [57]. Higher peak values for *G′* and *G″* of the samples with dairy ingredients compared to the control can probably be due to the gelation of dairy proteins induced by the heat process. 

### 4.2. Bread Characteristics

The specific volume of the tested samples was lower compared to the control, but among the samples with dairy ingredients, a higher decrease was observed in the case of water replacement by milk. Bread volume is known to be negatively affected by acid casein, whey proteins and lactose [25,79]. Water soluble or insoluble components addition, e.g., whey or milk, may cause water activity changes that influence dough physical characteristics, causing its stiffening, since the interactions between starch grains and other ingredients’ components depend on the water amount [79]. Some of the water soluble particles may decrease the number of contact points of the continuous phase, leading to dough rheological changes, depending on the flour characteristics and available water and consequently varying the specific volume of bread produced under fixed conditions [67,79]. Lipids’ presence in the dough system can affect bread volume and crumb structure, as non-polar lipids seem to destabilize gas cells and decrease final product volume [80,81]. A lower porosity of the samples with dairy ingredients was similar to the results obtained by Zhou et al. [72] for bread enriched with whey protein and can be due to the gelation capacity of dairy proteins, which lead to higher viscosity. The decrease of elasticity with acid whey level increase can probably be attributed to the interactions of whey protein with the gluten matrix by structure weakening effect and to the lactose and mineral elements like sodium and potassium dough strengthening effect, respectively [12]. The significant (*p* < 0.01) positive correlation between specific volume and recovery compliance means that dough with higher recovery capacity is associated with larger bread volume, which is in agreement with the results obtained by Wang and Sun [21]. This probably suggests that the recovery strain represents the elastic characteristic of dough, for bread baking performance dough being required to have both viscous and elastic character [82]. The significant (*p* < 0.05) negative correlation of bread volume with the retardation time in the creep phase (*λ_C_*) is consistent with those previously reported in the literature [22], indicating that dough with faster recovery will give bread with lower volume.

Firmness, gumminess, chewiness and resilience values increase when water was replaced by dairy ingredients and are in agreement with the results obtained for wheat and wheat-rye bread with acid whey addition [12]. Bread firmness is influenced by the surface viscosity of the lamellar liquid from the structure, so milk or whey containing samples firmness increases can be due to the higher surface viscosity, which gives firmer and more stable structures [83]. Therefore, the interactions between dairy ingredients proteins and starch increased the viscosity of the lamellar liquid which determined firmer bread crumb. Higher gumminess can be attributed to the gluten weakening effect of whey and milk components which lead to a more compact dough network [12]. Calcium ions present in dairy ingredients may produce gluten network dehydration and lower the water amount osmotically linked which conduct to strengthen and more compact dough structure [84]. Starch granules are affected by the addition of non-starch ingredients and thus the mechanical properties of bread are changed, as the gas cell walls contain starch granules that, in lower amounts, give thinner structures [85]. Thinner cell walls can be associated with higher protein fractions strain hardening, leading to greater bread stiffness and strength [85,86]. Crumb firmness positive correlation indicates that high bread firmness is associated with high dough elastic and complex flow indices; similar results being obtained by Demirkesen et al. [87].

Bread crumb microstructure is of great importance for consumer’s choice, as baked products with uniform and high porosity are desired. Bread crumb can be considered an anisotropic open-cell cellular solid material with a large range of pores sizes, its structure being hierarchically organized in both void and solid phases [88]. Pores density and size increased for milk containing samples and decreased for those with acid whey, which is in agreement with the literature, the presence of lactic acid bacteria influencing the fermentation process, and consequently, the pores formation [89]. The gluten network can be negatively influenced by the acids produced by the lactic bacteria, which led to gas retention reduction during the proofing stage, and thus lower bread porosity [90]. The differences of pores sizes and densities between milk and acid whey containing samples can possibly be caused by the pH differences between these two ingredients, since pH value significantly influences yeast activity and consequently bread pores structure [90]. Crumb structure is expected to be influenced by the way in which the liquid phase is spread in the gas cell walls during proofing and baking [85]. Kenny et al. [26] showed the relation between bread visual microstructure and dough rheology, underlying that low storage and loss moduli can be due to the less compact protein network, our results for whey containing samples, W2 and W3, being in agreement with that. Both microstructural and rheological characteristics are influenced by the water available in the dough system, its interactions with other components and its mobility [91]. 

Generally, an increase of the sensory characteristics scores was obtained for the samples with milk or acid whey, bread appearance, color and texture being significantly different (*p* < 0.05) between W1 and W2 samples, while no significant differences (*p* > 0.05) were observed when milk was added. The flavor, smell, taste and acceptability characteristics of bread were not significantly different (*p* > 0.05) among the studied samples. An increase of the sensory characteristics of baladi bread was reported by Gaber et al. [92], due to the content of organic acids, lactose and salts of the acid whey. Improved sensory characteristics were also obtained by Kakan et al. [93] for milk bread and by Paul et al. [4] for multigrain bread, with water replacement by Paneer whey. Our results, showing no statistically significant differences compared to the control, can probably be due to the lower amounts of dairy ingredients compared to those used in other studies.

## 5. Conclusions

The replacement of water with milk or acid whey influenced dough rheological characteristics and final product quality, as a function of the level and dairy ingredient type used. Compared to the control, the storage and loss moduli increased when milk substituted water and decreased when acid whey was used. The resistance to deformation decreased at more than 25% replacement level by milk and increased for samples with up to 25% replacement by acid whey. The maximum gelatinization temperatures increased for both milk and acid whey containing dough samples. A decrease of bread physical characteristics was obtained as a function of the replacement level and compared to the control, except the elasticity, which was improved. Milk and acid whey containing samples presented higher crumb firmness, gumminess, chewiness and resilience compared to the control. At more than 25%, the replacement of water with acid whey determined a decrease of pores density and size compared to the control and with the level increase, while the opposite trend was observed for milk containing samples. High sensory characteristics scores were obtained for samples with more than 25% milk and acid whey. This study revealed essential information about the possibility of using milk or acid whey as water substitutes in specialty bread making, and can be helpful for further optimizations. The use of dairy ingredients in this way leads to product diversity and better local resources management. However, it should be mentioned that the results presented are at a pilot scale, industrial production evaluation being needed. 

## Figures and Tables

**Table 1:** Power law models parameters describing the dependence of storage, loss and complex moduli on frequency.

Sample	$G^{\prime }\left(\omega \right)=K^{\prime }\cdot \omega^{n^{\prime }}$		$G^{\prime \prime }\left(\omega \right)=K^{\prime \prime }\cdot \omega^{n^{\prime \prime }}$		$G^{*}\left(\omega \right)=K^{*}\cdot \omega^{n^{*}}$	
	*K′* (Pa·s^n′^)	*n′*	*K″* (Pa·s^n″^)	*n″*	K* (Pa·s^n″^)	n*
**C**	67,170.67 ± 59.980 ^bw^	0.168 ± 0.002 ^ax^	18,873.054 ± 100.052 ^aw^	0.251 ± 0.020 ^ax^	69,705.540 ± 24.000 ^bw^	0.176 ± 0.009 ^ax^
**M1**	60,297.61 ± 100.014 ^a^	0.197 ± 0.006 ^b^	18,996.005 ± 60.005 ^a^	0.266 ± 0.004 ^a^	63,073.670 ± 20.015 ^a^	0.206 ± 0.011 ^b^
**M2**	81,740.20 ± 70.710 ^d^	0.209 ± 0.007 ^b^	28,679.779 ± 20.024 ^c^	0.262 ± 0.002 ^a^	86,570.750 ± 100.100 ^d^	0.216 ± 0.009 ^b^
**M3**	72,493.27 ± 79.980 ^c^	0.206 ± 0.003 ^b^	25,774.818 ± 99.900 ^b^	0.255 ± 0.003 ^a^	76,866.193 ± 30.102 ^c^	0.213 ± 0.003 ^b^
One-way ANOVA *p* values						
	*p* < 0.001	*p* < 0.001	*p* < 0.001	ns	*p* < 0.001	*p* < 0.001
**W1**	48,054.22 ± 29.793 ^z^	0.216 ± 0.005 ^y^	18,251.391 ± 39.918 ^z^	0.256 ± 0.001 ^x^	51,381.573 ± 9.239 ^z^	0.222 ± 0.019 ^y^
**W2**	39,534.26 ± 50.029 ^x^	0.218 ± 0.000 ^y^	14,928.972 ± 70.016 ^x^	0.247 ± 0.003 ^x^	42,250.319 ± 100.170 ^x^	0.223 ± 0.009 ^y^
**W3**	42,774.78 ± 100.129 ^y^	0.232 ± 0.001 ^z^	16,458.544 ± 159.740 ^y^	0.268 ± 0.006 ^x^	45,813.940 ± 98.494 ^y^	0.237 ± 0.003 ^y^
One-way ANOVA *p* values						
	*p* < 0.001	*p* < 0.001	*p* < 0.001	ns	*p* < 0.001	ns

Milk containing samples: a–d, mean values in the same column followed by different letters are significantly different (*p* < 0.05); whey containing samples: w–z, mean values in the same column followed by different letters are significantly different (*p* < 0.05); ns: not significant; C—control sample; M1—12.5% milk; M2—25% milk; M3—50% milk; W1—12.5% acid whey; W2—25% acid whey; W3—50% acid whey; *G′*—storage modulus (Pa); *G″*—loss modulus (Pa); G*—complex modulus (Pa); *ω*—angular frequency (rad/s); *K′*, *K″*, K* (Pa·s^n′^)—consistency indices; *n′*, *n″*, n*—flow behavior indices.

**Table 2:** Parameters of creep-recovery modeling, Equations (5) and (6) and gelatinization temperatures.

Sample	Creep Phase					Recovery Phase					Temperature Sweep
	*J_Co_·10^5^* (Pa^−1^)	*J_Cm_·10^5^* (Pa^−1^)	*λ_C_* (s)	*μ_Co_·10^−6^* (Pa s)	*J_max_·10^5^* (Pa^−1^)	*J_Ro_·10^5^* (Pa^−1^)	*J_Rm_·10^5^* (Pa^−1^)	*λ_R_* (s)	*J_r_·10^5^* (Pa^−1^)	*J_r_/J_max_* (%)	*T_max_* (°C)
**C**	4.21 ± 0.03 ^cy^	10.01 ± 0.01 ^cx^	30.77 ± 0.07 ^ax^	1.03 ± 0.02 ^bz^	14.96 ± 0.16 ^by^	2.85 ± 0.02 ^bx^	6.70 ± 0.04 ^dz^	49.26 ± 0.02 ^cw^	9.55 ± 0.06 ^cy^	63.84 ± 0.28 ^ax^	84.70 ^ax^
**M1**	5.17 ± 0.02 ^d^	10.00 ± 0.01 ^c^	32.57 ± 0.09 ^c^	0.88 ± 0.01 ^a^	17.63 ± 0.30 ^c^	5.86 ± 0.06 ^d^	6.10 ± 0.02 ^c^	40.48 ± 0.04 ^b^	11.96 ± 0.07 ^d^	67.84 ± 0.70 ^a^	87.70 ^b^
**M2**	2.90 ± 0.06 ^b^	8.61 ± 0.02 ^b^	32.05 ± 0.03 ^b^	1.52 ± 0.01 ^c^	10.40 ± 0.09 ^a^	2.19 ± 0.07 ^a^	4.69 ± 0.06 ^b^	51.02 ± 0.07 ^d^	6.88 ± 0.13 ^b^	66.15 ± 4.51 ^a^	87.70 ^b^
**M3**	2.51 ± 0.08 ^a^	8.10 ± 0.03 ^a^	33.67 ± 0.06 ^d^	1.51 ± 0.01 ^c^	9.45 ± 0.10 ^a^	3.19 ± 0.08 ^c^	3.40 ± 0.05 ^a^	34.48 ± 0.09 ^a^	6.59 ± 0.13 ^a^	69.74 ± 0.64 ^a^	87.70 ^b^
One-way ANOVA *p* values											
	*p* < 0.001	*p* < 0.001	*p* < 0.001	*p* < 0.001	*p* < 0.001	*p* < 0.001	*p* < 0.001	*p* < 0.001	*p* < 0.001	ns	*p* < 0.001
**W1**	7.27 ± 0.02 ^w^	20.00 ± 0.02 ^y^	30.76 ± 0.06 ^x^	0.68 ± 0.02 ^x^	23.81 ± 0.09 ^w^	6.76 ± 0.03 ^y^	8.82 ± 0.02 ^w^	43.10 ± 0.05 ^y^	15.58 ± 0.05 ^w^	65.43 ± 0.04 ^y^	93.80 ^z^
**W2**	4.80 ± 0.06 ^z^	20.01 ± 0.01 ^y^	37.03 ± 0.07 ^z^	0.75 ± 0.03 ^y^	18.39 ± 0.08 ^z^	8.66 ± 0.01 ^z^	4.94 ± 0.05 ^x^	28.08 ± 0.02 ^x^	13.60 ± 0.06 ^z^	73.95 ± 0.00 ^w^	90.80 ^y^
**W3**	3.73 ± 0.05 ^x^	10.01 ± 0.01 ^x^	31.44 ± 0.05 ^y^	1.18 ± 0.01 ^w^	13.15 ± 0.06 ^x^	2.82 ± 0.02 ^x^	5.86 ± 0.04 ^y^	45.66 ± 0.07 ^z^	8.68 ± 0.06 ^x^	66.03 ± 0.12 ^z^	90.80 ^y^
One-way ANOVA *p* values											
	*p* < 0.001	*p* < 0.001	*p* < 0.001	*p* < 0.001	*p* < 0.001	*p* < 0.001	*p* < 0.001	*p* < 0.001	*p* < 0.001	*p* < 0.001	*p* < 0.001

Milk containing samples: a–d, mean values in the same column followed by different letters are significantly different (*p* < 0.05); whey containing samples: w-z, mean values in the same column followed by different letters are significantly different (*p* < 0.05); ns: not significant; C—control sample; M1—12.5% milk; M2—25% milk; M3—50% milk; W1—12.5% acid whey; W2—25% acid whey; W3—50% acid whey; *J_Co_, J_Ro_* (Pa^−1^—instantaneous compliances for creep and recovery phase respectively; *J_Cm_*, *J_Rm_* (Pa^−1^)—retarded elastic compliances for creep an recovery phase respectively; *λ_c_*, *λ_R_* (s)—retardation times; *μ_Co_* (Pa·s)—zero shear viscosity; *J_max_* (Pa^−1^)—maximum creep compliance at the end of the creep test; *J_r_* (Pa^−1^)—recovery compliance; *T_max_*—maximum gelatinization temperature obtained at the peak of the temperature sweep test.

**Table 3:** Physical properties of bread with different water replacement levels.

Sample	Specific Volume (cm^3^/100 g)	Porosity (%)	Elasticity (%)
**C**	356.41 ± 7.02 ^cz^	77.23 ± 0.13 ^cw^	90.63 ± 0.01 ^cx^
**M1**	292.79 ± 5.63 ^b^	70.39 ± 0.10 ^a^	83.78 ± 0.03 ^a^
**M2**	281.89 ± 3.31 ^ab^	71.76 ± 0.13 ^b^	94.12 ± 0.02 ^d^
**M3**	273.77 ± 3.03 ^a^	70.31 ± 0.26 ^a^	88.57 ± 0.13 ^b^
One-way ANOVA *p* values			
	*p* < 0.001	*p* < 0.001	*p* < 0.001
**W1**	333.73 ± 0.40 ^y^	73.30 ± 0.05 ^x^	96.97 ± 0.17 ^w^
**W2**	306.87 ± 3.28 ^x^	73.70 ± 0.02 ^y^	94.30 ± 0.03 ^z^
**W3**	326.6 ± 4.57 ^y^	75.79 ± 0.02 ^z^	91.43 ± 0.01 ^y^
One-way ANOVA *p* values			
	*p* < 0.001	*p* < 0.001	*p* < 0.001

Milk containing samples: a–d, mean values in the same column followed by different letters are significantly different (*p* < 0.05); whey containing samples: w–z, mean values in the same column followed by different letters are significantly different (*p* < 0.05); ns: not significant. C—control sample, M1—12.5% milk, M2—25% milk, M3—50% milk, W1—12.5% acid whey, W2—25% acid whey, W3—50% acid whey.

**Table 4:** Texture parameters of bread with different water replacement levels.

Sample	Firmness (N)	Cohesiveness (Adimensional)	Gumminess (N)	Chewiness (J)	Resilience (Adimensional)
**C**	5.26 ± 0.45 ^ax^	0.73 ± 0.03 ^ay^	3.86 ± 0.47 ^ax^	3.86 ± 0.46 ^ax^	1.36 ± 0.14 ^ay^
**M1**	7.93 ± 0.53 ^b^	0.80 ± 0.01 ^b^	6.38 ± 0.49 ^b^	6.37 ± 0.49 ^b^	1.89 ± 0.21 ^b^
**M2**	11.07 ± 1.85 ^c^	0.74 ± 0.04 ^a^	8.19 ± 1.25 ^c^	8.19 ± 1.25 ^c^	1.53 ± 0.16 ^ab^
**M3**	9.79 ± 0.59 ^bc^	0.77 ± 0.02 ^ab^	7.57 ± 0.29 ^bc^	7.56 ± 0.29 ^bc^	1.71 ± 0.22 ^ab^
One-way ANOVA *p* values					
	*p* < 0.001	*p* < 0.05	*p* < 0.001	*p* < 0.001	*p* < 0.05
**W1**	10.16 ± 1.28 ^y^	0.71 ± 0.07 ^y^	7.15 ± 0.44 ^y^	7.16 ± 0.43 ^y^	1.42 ± 0.31 ^y^
**W2**	11.75 ± 2.95 ^y^	0.66 ± 0.02 ^y^	7.67 ± 1.82 ^y^	7.67 ± 1.82 ^y^	1.41 ± 0.06 ^y^
**W3**	10.92 ± 1.34 ^y^	0.55 ± 0.04 ^x^	5.97 ± 0.54 ^y^	6.04 ± 0.66 ^y^	0.88 ± 0.13 ^x^
One-way ANOVA *p* values					
	*p* < 0.001	*p* < 0.001	*p* < 0.001	*p* < 0.001	*p* < 0.05

Milk containing samples: a–c, mean values in the same column followed by different letters are significantly different (*p* < 0.05); whey containing samples: x and y, mean values in the same column followed by different letters are significantly different (*p* < 0.05); ns: not significant. C—control sample, M1—12.5% milk, M2—25% milk, M3—50% milk, W1—12.5% acid whey, W2—25% acid whey, W3—50% acid whey.

**Table 5:** Computed bread crumb features.

Sample	Pores Density (1/cm^2^)	Mean Cell Size (mm^2^)	Pore Circularity (Adimensional)	Cell Area Fraction (%)
**C**	30.04 ± 1.77 ^by^	0.30 ± 0.05 ^abx^	0.26 ± 0.05 ^ax^	27.12 ± 2.02 ^ax^
**M1**	20.42 ± 1.55 ^a^	0.20 ± 0.02 ^a^	0.17 ± 0.03 ^a^	30.46 ± 1.77 ^a^
**M2**	35.60 ± 3.87 ^b^	0.35 ± 0.04 ^b^	0.16 ± 0.05 ^a^	38.21 ± 7.80 ^a^
**M3**	32.35 ± 1.03 ^b^	0.32 ± 0.07 ^ab^	0.20 ± 0.06 ^a^	35.21 ± 4.54 ^a^
One-way ANOVA *p* values				
	*p* < 0.001	*p* < 0.05	ns	ns
**W1**	30.00 ± 4.07 ^y^	0.30 ± 0.11 ^x^	0.19 ± 0.06 ^x^	35.13 ± 5.01 ^x^
**W2**	19.06 ± 1.07 ^x^	0.19 ± 0.05 ^x^	0.29 ± 0.07 ^x^	28.75 ± 3.23 ^x^
**W3**	21.45 ± 0.91 ^x^	0.22 ± 0.02 ^x^	0.19 ± 0.03 ^x^	30.42 ± 3.99 ^x^
One-way ANOVA *p* values				
	*p* < 0.001	ns	ns	ns

Milk containing samples: a and b, mean values in the same column followed by different letters are significantly different (*p* < 0.05); whey containing samples: x and y, mean values in the same column followed by different letters are significantly different (*p* < 0.05); ns: not significant. C—control sample, M1—12.5% milk, M2—25% milk, M3—50% milk, W1—12.5% acid whey, W2—25% acid whey, W3—50% acid whey.

**Table 6:** Correlations between dough and bread characteristics.

	Volume	Firmness	*K′*	*n′*	*K″*	*n″*	K*	n*	*J_Co_*	*J_Cm_*	*λ_C_*	*μ_Co_*	*J_max_*	*J_Ro_*	*J_Rm_*	*λ_R_*	*J_r_*	*J_r_/J_max_*	Pores Density	T_max_
**Volume**	1.00	ns	ns	ns	−0.63 **	ns	−0.45 *	ns	0.50 *	ns	−0.46 *	−0.54 *	0.50 *	ns	0.74 **	ns	ns	−0.49 *	ns	ns
**Firmness**		1.00	ns	0.75 **	ns	ns	ns	0.61 **	ns	ns	ns	ns	ns	ns	ns	ns	ns	ns	ns	0.64 **
***K′***			1.00	−0.52 *	0.91 **	ns	0.99 **	−0.46 *	−0.58 **	−0.71 **	ns	0.74 **	−0.64 **	−0.68 **	ns	0.46 *	−0.70 **	ns	0.75 **	−0.71 **
***n′***				1.00	ns	ns	−0.48 *	0.91 **	ns	ns	ns	ns	ns	ns	ns	ns	ns	ns	ns	0.81 **
***K″***					1.00	ns	0.93 **	ns	−0.60 **	−0.61 **	ns	0.83 **	−0.67 **	−0.62 **	−0.50 *	ns	−0.70 **	ns	0.79 **	−0.42 *
***n″***						1.00	ns	ns	ns	ns	ns	ns	ns	ns	ns	ns	ns	ns	−0.10	ns
K*							1.00	ns	−0.59 **	−0.71 **	ns	0.76 **	−0.65 **	−0.68 **	ns	0.45 *	−0.70 **	ns	0.76 **	−0.69 **
n*								1.00	ns	ns	ns	ns	ns	ns	ns	ns	ns	ns	ns	0.72 **
***J_Co_***									1.00	0.77 **	ns	−0.90 **	0.99 **	0.70 **	0.88 **	ns	0.95 **	ns	ns	0.56 **
***J_Cm_***										1.00	ns	−0.81 **	0.84 **	0.87 **	0.52 *	−0.47 *	0.89 **	ns	ns	0.71 **
***λ_C_***											1.00	ns	ns	0.58 **	−0.57 **	−0.87 **	ns	0.89 **	ns	ns
***μ_Co_***												1.00	−0.95 **	−0.83 **	−0.72 **	ns	−0.96 **	ns	0.56 **	−0.46 **
***J_max_***													1.00	0.78 **	0.83 **	ns	0.98 **	ns	ns	0.57 **
***J_Ro_***														1.00	ns	−0.71 **	0.88 **	0.55 **	−0.57 **	0.56 **
***J_Rm_***															1.00	ns	0.72 **	−0.51 *	ns	ns
***λ_R_***																1.00	ns	−0.83 **	0.48 *	ns
***J_r_***																	1.00	ns	−0.46 *	0.60 **
***J_r_/J_max_***																		1.00	ns	ns
**Pores density**																			1.00	ns
**T_max_**																				1.00

* *p* < 0.05; ** *p* < 0.01; ns-not significant; Abbreviations used for the correlated parameters are presented in the Materials and methods section.
